# A study on the relationship between technological affordance of age-appropriate smart wearable devices and the well-being of older adults

**DOI:** 10.3389/fpubh.2025.1672136

**Published:** 2025-11-11

**Authors:** Hao Zhang, Shusheng Yang, Yili Lin

**Affiliations:** 1 School of Humanities and Foreign Languages, Qingdao University of Technology, Qingdao, China; 2 School of Economics and Management, Beijing University of Technology, Beijing, China

**Keywords:** age-appropriate wearable electronic devices, aged, technological affordance theory, psychological needs, well-being

## Abstract

**Introduction:**

Global population aging is intensifying, posing significant challenges to care for older adults. In this context, age-appropriate smart wearable devices, with their capabilities for efficient physiological monitoring, disease prevention, and safety protection, play an important role in supporting the health and independence of older adults. However, the relationship between the technological affordance of these devices and the well-being of older adults has not been fully explored. In this study, an integrated theoretical model was constructed based on Technological Affordance Theory (TAT), Self-Determination Theory (SDT), Stimulus-Organism-Response (SOR) theory, and Socioemotional Selectivity Theory (SST).

**Methods:**

Data from 233 older adults using age-appropriate smart wearable devices were collected through a questionnaire, and structural equation modeling (SEM) was used to analyze the data.

**Results:**

The results indicate that the visibility (*β* = 0.155–0.238, *p* < 0.05), interactivity (*β* = 0.155–0.304, *p* < 0.05), and directiveness (*β* = 0.292–0.395, *p* < 0.001) of age-appropriate smart wearable devices are significantly and positively correlated with older adults’ sense of autonomy, competence, and relatedness. Furthermore, these psychological needs are significantly and positively correlated with their overall well-being (*β* = 0.184–0.352, *p* < 0.01). Sense of autonomy, competence, and relatedness significantly mediated the relationship between technological affordance and older adults’ well-being (indirect effects = 0.034–0.139, 95% confidence interval [CI] excluding 0).

**Discussion:**

This study reveals that technological affordance is indirectly positively correlated with the well-being of older adults via their psychological needs, offering practical insights for the design and development of age-appropriate smart wearable devices.

## 1 Introduction

According to the United Nations World Population Prospects 2022, the global population aged 65 and over is expected to increase from 761 million in 2021 to 1.6 billion in 2050, making healthy aging a global concern (1). Against this backdrop, China’s average life expectancy has risen to 78 years; however, population ageing is accelerating due to a continued decline in birth rates (2). According to data released by the National Bureau of Statistics in 2023, the population aged 65 and above in China has reached 217.09 million, accounting for 15.4 percent of the total national population, marking the country’s entry into a moderately aging society (3). To meet this challenge, the Chinese government is actively exploring an aging model that suits the country’s situation, while focusing on digital information technology-enabled healthy aging (1). As a product of the digital age, age-appropriate smart wearable devices play an important role in care for older adults with their functions of efficient physiological data collection, disease prevention and safety protection, significantly reducing the burden of care on families and society (4). Therefore, it is of great significance to conduct in-depth research on the application of age-appropriate smart wearable devices in the field of geriatric services.

However, today’s development of aging-appropriate smart wearable devices is seriously competitive in the market, overly focused on the development of product sales channels and market share, and the development of products with many functions but not refined, focusing on a single direction, ignoring the connection between technological affordance and the real feelings of older adults and even the sense of well-being (5). In addition, most of the current research is focused on the development of new technologies, new functions (6), and few overall studies have been found that link the visibility, interactivity, and directiveness of the technological affordance of age-appropriate smart wearable devices to the well-being of older adults and make a collection and analysis of data from specific samples. All these status quo situations are detrimental to the development of the care industry for older adults and even to the long-term development of healthy ageing.

In order to investigate the relationship between the technological affordance of age-appropriate smart wearable devices and the well-being of older adults, and to promote the flourishing development of smart aging, this paper draws on TAT, SDT, SOR theory, and SST. It examines how the visibility, interactivity, and directiveness of technological affordance relate to three psychological needs of older adults: the sense of autonomy, the sense of competence, and the sense of relatedness. Furthermore, the study also explores the connection between these psychological needs and well-being. Through the above exploration, the study reveals the close connection between technological affordance and the well-being of older adults. This study enriches the application of the above four theories in the context of age-appropriate smart wearable devices, and bridges the deficiency in exploring the well-being of older adults by integrating TAT with SOR theory, SDT, and SST. This study also innovatively proposes the design principle of “psychological needs orientation,” which integrates the sense of autonomy, competence, and relatedness from SDT into the design of age-appropriate smart wearable devices, breaking through the limitations of traditional functional needs and providing a new theoretical framework for the field of smart aging. This principle emphasizes the psychological needs of older adults as the core, and enhances their experience of use and sense of well-being by optimizing technical interaction and functional design. In practice, this study provides practical solutions for the research and development of smart aging products, promotes the profound transformation of ageing-friendly devices from functional-oriented to human-oriented, and provides important practical insights for the design and development of ageing-friendly smart devices.

## 2 Theoretical foundation

### 2.1 Age-appropriate smart wearable devices

Smart wearable devices are smart devices that can collect health data, conduct health assessments, and provide decision support. They achieve these functions through various embedded components (such as sensors, actuators, and smart fabrics) and are supported by technologies including wireless sensor networks (7). Current smart wearable devices, such as smartwatches, health trackers, smart glasses, and smart clothing, have applications focused on vital signs monitoring, physical activity assessment, positioning, and fall monitoring and prevention (8). In this study, the scope of “age-appropriate” is defined as smart wearable devices designed for community-dwelling older adults who experience common, mild physical or cognitive impairments associated with normal aging. This definition specifically focuses on the majority of older users who maintain functional independence but benefit from technological support to compensate for age-related declines. It explicitly excludes designs for individuals with moderate to severe disabilities who require specialized medical or assistive devices. In the context of population aging, age-appropriate smart wearable devices refer to intelligent electronic devices worn on the body or integrated into clothing, specifically optimized for the physiological characteristics, psychological needs, and usage habits of older adults (9). These devices include smart emergency call bracelets, smartwatches with fall detection and health monitoring capabilities, and anti-wandering smart location trackers. Their primary goal is to leverage technologies such as the Internet of Things, big data, and artificial intelligence to enhance older adults’ quality of life, health, and safety. Specifically, they can help older adults overcome obstacles in cognitive ability, mobility, and psychosocial functioning, as well as obtain instant feedback on vital signs such as heart rate, blood pressure, and blood glucose, among others. At the same time, these devices are designed with a low learning threshold to ensure technology is accessible and beneficial to older adults (9). Age-appropriate smart wearable devices also play a significant role in enhancing older adults’ Activities of Daily Living (ADL). ADL refers to the essential activities that individuals repeatedly perform daily to meet their basic needs in daily life. They reflect the core ability of individuals to maintain independent living in families, medical institutions, or communities, and cover two major categories: basic self-care activities (such as eating, bathing, dressing, etc.) and instrumental activities (such as shopping, meal preparation, transportation, etc.) (10). In addition, age-appropriate smart wearable devices can also help healthcare professionals collect health data of older adults more efficiently and accurately and make timely diagnosis, and improve the efficiency of doctor-patient communication. It can also provide personalized health management to improve older adults’ quality of life. For example, smartwatches equipped with fall detection algorithms can automatically summon emergency services, thus reducing the risks associated with prolonged immobility. Meanwhile, wearable sensor patches for continuous blood glucose monitoring can track metabolic parameters in real time, alert users to potential abnormalities, and empower them to make proactive dietary adjustments (11). Recent studies have shown that older adults are increasingly interested in using digital technology to manage their health, such as stress and heart rate measurements and activity diaries (12). This sets the stage for the adoption of age-appropriate smart wearables. However, most previous research has prioritized the multifunctional development of age-appropriate smart wearables and the design of simplified human-computer interfaces (13). This focus has overlooked an exploration of the close relationship between technology and the physiological and psychological needs of older adults from the perspectives of visibility, interactivity, and directiveness of technological affordance. Furthermore, there has been a lack of collection, summarization, and analysis of relevant real-life data. To utilize wearable devices to facilitate the realization of healthy aging, it is necessary to gain a deeper understanding of the inner feelings and practical needs of older adults for age-appropriate smart wearable devices. Research demonstrates that older users’ technology adoption is strongly influenced by their perception of ease of use, with most expressing confidence in learning to use wearables when provided with appropriate support (14). From a design perspective, devices must accommodate age-related changes through enhanced screen visibility and voice interaction capabilities (15). Furthermore, studies indicate that older adults show greater concern about device integration into daily routines than data accuracy, highlighting the importance of compatibility and ongoing usage support (16). Therefore, we explore the connection between smart wearable devices and the well-being of older adults from the perspective of the technological affordance of age-appropriate smart wearable devices.

### 2.2 Self-determination theory

SDT provides a framework for the study of human motivation by assuming that people are naturally active and motivated to grow, learn, and change, and by categorizing types of motivation from controlled to autonomous, as well as amotivation (17). This foundational distinction between intrinsic motivation (driven by inherent satisfaction in an activity) and extrinsic motivation (driven by external contingencies like rewards or punishment) was first systematically articulated by Deci and Ryan in (18), who also identified the social environment as a critical factor in nurturing intrinsic motivation. This line of thinking was later expanded by Ryan and Deci in (19), who emphasized that satisfaction of basic psychological needs is a universal driver of psychological growth and well-being across diverse domains—including health management and technology use, which are directly relevant to older adults’ engagement with age-appropriate wearables. This is corroborated by meta-analytic findings, which confirm that interventions supporting basic psychological needs indeed promote autonomous motivation and facilitate health behavior change across various populations (20). The distinction between these motivational types is crucial, as validated by research on health behaviors showing that autonomous regulation is a more consistent and positive predictor of behavioral engagement than controlled regulation (21). The social environment in which an individual lives and the support of others in the community may reinforce or threaten the fulfillment of an individual’s psychological needs for a sense of autonomy, competence, and relatedness (17). If a person always feels a sense of autonomy, competence and relatedness in their daily work and life, then the individual will also naturally have a sense of well-being (22). When exploring the connection between SDT and older adults’ technology adoption, existing research has provided important empirical support for the theory’s applicability. Among such studies, the research by Trinh et al. (23) on Digital Health Portals (DHPs) is particularly representative: focusing on 26 older adults aged 60–85 who logged into DHPs at least twice a year, they found that their results were highly consistent with the core tenets of SDT. Specifically, when older adults chose to use DHPs voluntarily, the fulfillment of their autonomy need significantly enhanced their intrinsic motivation to use the technology; the development of participants’ sense of competence depended both on the user-friendly design of DHPs themselves and the older adults’ mastery of basic operational skills; meanwhile, the relatedness need was effectively strengthened through encouragement from healthcare providers and assistance from family members. This study not only highlights the value of SDT in explaining older adults’ adoption behaviors of digital health technologies but also reveals the critical link between psychological needs and technology design, providing reliable reference for the application of SDT in subsequent research. Based on SDT, this paper starts from the three dimensions of sense of autonomy, competence, and relatedness, using them as mediating variables to explore how technological affordance establishes a close relationship with the well-being of older adults through these three-dimensional needs. Sense of autonomy refers to the fact that the care and other functions of age-appropriate smart wearable devices can make older adults feel more independent and that older adults can decide independently which function to use and how to use it according to their own principles or priorities in the process of using age-appropriate smart wearable devices, and all choices and actions are voluntary, which strengthens older adults’ sense of autonomy (24). Sense of competence refers to older adults’ belief in their ability to use an age-appropriate smart wearable device and its features as well as their ability to accomplish a task with it. Sense of competence makes older people feel more relaxed in the process of using the smart wearable device and makes them feel that they have a sense of positive interaction with the people, things and objects around them after using the age-appropriate smart wearable device (25). A sense of relatedness refers to the fulfillment of the relational needs of older people by age-appropriate smart wearables. This allows older persons to feel connected to the world and to others, increasing their sense of belonging (26).

### 2.3 Technological affordance theory

Affordance theory initially conceptualized affordance as the “possibilities for action” that an environment provides to individuals. This core definition was first proposed by Gibson (27), who emphasized that affordances are objective properties of the environment, yet perceived and acted upon based on an individual’s physical capabilities and goals (e.g., a chair “affords sitting” for most people). In human-computer interaction and information systems research, technological affordance denotes the action opportunities that information and communication technologies (ICT) offer users (28). In addition, Markus and Silver state that technological affordance represents the possibilities for goal-oriented action that technological objects offer users (88), and that it provides a clear explanation of how and why the same technology can be used or produce different results in different situations (29). The theory is commonly used in research on the design of products and services and their sustainability (30), fitting the purpose of the study explored in this paper on the close relationship of technological affordance with the well-being of older adults in the context of the application of age-appropriate smart wearable devices. Recent work has refined the application of affordance theory to smart wearables by linking device features to age-specific behavioral mechanisms: studies note that “health data affordance”—enabled by real-time monitoring of vital signs—supports older adults’ autonomous health management only when paired with simplified interpretation tools (e.g., color-coded alerts), as cognitive declines may hinder complex data analysis (5); meanwhile, “social connection affordance” (e.g., shared activity challenges) is particularly impactful for reducing loneliness in this group, but its effectiveness depends on aligning interaction frequency with older adults’ preference for low-demand social engagement (16), and these nuanced findings not only advance theoretical understanding of affordance in gerontechnology but also provide targeted insights for how we conceptualize the technological affordance dimensions of age-appropriate smart wearables below. Age-appropriate smart wearable devices greatly facilitate the lives of seniors in their later years by providing them with health detection, vital signs monitoring, medical care, fall prevention, safety alerts, as well as entertainment and social connections (31). Therefore, based on the functions of age-appropriate smart wearable devices and the needs of older adults, this paper divides the technological affordance into three dimensions: visibility, interactivity and directiveness. Visibility, which refers to the visualization of the aging smart wearable device’s exclusive device interface for older adults as well as the intuitive and clear data content and presentation, thus giving older adults an intuitive visual experience (32). This “intuitive visual experience” means the interface is immediately understandable without requiring extensive learning or prior technical knowledge, leveraging familiar visual cues (e.g., using a heart icon for heart rate, large and clear fonts) and simplified layouts tailored to age-related changes in perception and cognition, thus enabling older users to grasp information effortlessly and interact with the device confidently (33). Interactivity, which refers to the human-computer interaction of age-appropriate smart wearable devices as well as social interaction between people (including daily communication with friends and relatives, sharing data information of wearable devices to doctors, etc.) and other functions (34). Directiveness, which refers to the user guide for the device in question, as well as the design of pathways or hints within the device, etc. (35). The three dimensions align closely with older adults’ core needs: visibility addresses their perceptual and cognitive declines (e.g., presbyopia) through intuitive interfaces and visualized data, enabling autonomous data understanding (36). Interactivity fits their emotional priorities (rooted in SST) and limited dexterity—voice interaction eases use, while social features like data sharing boost relatedness (37). Directiveness reduces digital anxiety via real-time guidance, enhancing operational confidence and competence (38). All map to the psychological needs in SDT, justifying their selection.

### 2.4 Stimulus-organism-response theory

SOR theory was developed from the classical stimulus–response (SR) theory of behaviorism, which describes the relationship between situational cues, an individual’s internal state, and subsequent behavior by analogizing them to stimuli, organisms, and responses, respectively. That is, situational cues influence a person’s behavior by altering his or her internal states (39). SOR theory is primarily used to explore the psychological effects of various external stimuli on an individual’s cognition or emotions and subsequent related behavioral responses (40). Previously, SOR theory has been widely used in research on the development and design of products and services (41). However, few studies have so far applied it to research on the relationship between the technological affordance of age-appropriate smart wearables and the well-being of older adults. To this end, this paper constructs a theoretical model based on the SOR paradigm to examine the relationship between the technological affordance of age-appropriate smart wearable devices and the well-being of older adults in the context of smart aging, specifically taking the technological affordance of age-appropriate smart wearable devices as stimulus (S), the psychological needs as organism (O), and the well-being of older adults as response (R).

### 2.5 Socioemotional selectivity theory

SST focuses on how individuals’ subjective perception of “future time” influences the priority of their social goals and behavioral choices, providing a supplementary theoretical perspective for understanding the psychological needs and technology acceptance logic of the older adult group in this study. The core view of SST holds that when individuals perceive future time as “limited” (a typical cognitive characteristic of older adults), they shift their social goal priorities from “knowledge acquisition goals” (such as expanding new social relationships and exploring unknown possibilities) to “emotional management goals” (such as deepening intimate relationships, avoiding negative emotions, and maximizing emotional value) (42). This goal shift is not a passive adaptation to aging-related declines but an active, adaptive strategy shaped by the subjective perception of time scarcity (42). Early foundational work on SST emphasized that this temporal framing of the future is a universal mechanism guiding motivational processes across adulthood, with older adults’ narrowed time horizons amplifying the salience of emotionally meaningful experiences over exploratory or knowledge-seeking activities (43). Subsequent refinements of the theory further clarified that this shift manifests in cognitive and behavioral patterns, such as increased attention to positive stimuli and reduced engagement with negative social interactions, as older adults prioritize maintaining emotional equilibrium (44). Moreover, the “social contraction” of older adults is not a result of cognitive or motor ability decline, but an active choice to concentrate their limited energy on relationships that can bring stable positive emotions (45). This selective investment in social ties aligns with SST’s prediction that time perception drives resource allocation: older adults filter social networks to retain connections that consistently fulfill emotional needs, rather than expending effort on peripheral relationships with uncertain emotional returns (42). For the older adult group focused on in this study, SST reveals their underlying demand characteristics: with increasing age, older adults’ psychological needs for “emotional connection,” “autonomous control,” and “ability recognition” are significantly enhanced, while they tend to avoid things that require high learning costs and offer no clear emotional returns (such as complex technical operations and unfamiliar social interactions) (46). Empirical evidence supporting SST has shown that this preference for emotional meaningfulness extends to information processing and decision-making—for instance, older adults exhibit better memory retention for emotionally positive information compared to negative or neutral content, a phenomenon linked to their prioritization of emotional goals (47). This demand characteristic is highly consistent with the design goal of age-appropriate smart wearable devices—through technical optimization to lower the usage threshold and accurate alignment with the emotional and psychological needs of older adults, these devices can truly serve to support the improvement of older adults’ well-being (46). In the context of this study, SST can provide logical support for explaining the internal connection between the technological affordance of age-appropriate smart wearable devices, the psychological needs of older adults (sense of autonomy, sense of competence, sense of relatedness), and well-being. The three dimensions of technological affordance (visibility, interactivity, directiveness) essentially align with the demand priorities of older adults from the perspective of SST through the optimization of technical features. This alignment with the aforementioned priorities coincides with the satisfaction of older adults’ psychological needs and the fulfillment of their emotional management goals, which in turn corresponds to the improvement of their well-being.

## 3 Research hypotheses

### 3.1 Technological affordance of age-appropriate smart wearable devices and psychological needs of older adults

Enhancing the technological affordance of age-appropriate smart wearables not only optimizes the healthcare process and facilitates the daily lives of older adults, but also significantly enhances their psychological functioning, which can ultimately be effective in motivating both older users and those suffering from geriatric diseases (28). In addition, studies have shown that older people can utilize wearable devices to gain a deeper understanding of their health status, which can increase their subjective willingness to take action to promote their health, thereby increasing their motivation to participate in their daily lives. Through the integration of medical and ergonomic knowledge as well as technological innovation, the technological affordance of age-appropriate smart wearable devices has been further enhanced, and they can play a role as daily assistive devices for older adults (48).

First, the visibility of age-appropriate smart wearable devices mainly includes data visualization and operation interface visualization (32). In terms of data visualization, user-centered data visualization for older adults can display the resulting data in the form of charts, graphs or information icons to help users quickly access and identify valid information (49). Currently, many data visualization techniques have been developed to effectively reduce mental effort and increase user understanding of data. For instance, simplified dynamic trend charts can intuitively present core health data of older adults, such as heart rate and sleep duration, on a daily/weekly basis. Combined with a color warning mechanism (e.g., the edge of the chart turns red when there is an abnormal heart rate), these charts enable users to quickly grasp their health status without complicated interpretation. Such lightweight visualization technologies have been widely applied in health monitoring devices for older adults (50). In terms of operator interface visualization, Shoemaker et al. (2010) propose a set of body-centered techniques that include “body-based tools” that are virtual tools at the waist or other locations that can be visualized on a display (89). The body-based data storage technology establishes a direct connection between the location of the user’s torso and the virtual container, and the data can be visualized on the display (51). Kim and Shin utilized a technology acceptance model incorporating psychological elements, including affective quality, relative advantage, mobility, usability, and subcultural appeal (90). Their analysis revealed that smartwatches with high mobility and visibility were perceived as easier to use, fostering a sense of autonomy and competence. Additionally, smartwatches exhibiting greater affective quality and relative advantage were viewed as offering higher perceived usefulness (52). In addition, by helping older persons to enhance their physical functions, participate in learning and engage in social activities in a visual way, such as through online video calls, age-appropriate smart wearable devices can significantly enhance their sense of autonomy, relatedness and belonging, reduce the burden of care on their families and society, and contribute to the harmony and development of society (48). Based on the above, we make the following assumptions:

H1a: Visibility of age-appropriate smart wearables is significantly positively correlated with older adults’ sense of autonomy.

H1b: Visibility of age-appropriate smart wearables is significantly positively correlated with older adults’ sense of competence.

H1c: Visibility of age-appropriate smart wearables is significantly positively correlated with older adults’ sense of relatedness.

Second, the interactivity of age-appropriate smart wearable devices mainly includes human–computer interaction and daily social activities between people (53). Human–computer interaction refers to the process of information exchange between humans and machines and systems. With the continuous development of science and technology, the form of human–computer interaction is constantly updated, and its main interaction methods include voice recognition, gesture recognition, tracking and so on. These new human–computer interaction methods greatly improve the efficiency and quality of interaction (54). Recently, the use of social compensation for social interactions among older adults has attracted significant attention, and more older adults are using social media under social networks to engage socially through an emerging medium, such as smart hand bands, smart watches, and other age-appropriate smart wearables (55). The interactivity of age-appropriate smart wearables can influence the sense of control and increase older adults’ behavior, which in turn affects the physicality of the interaction, while the sensory feedback presented may influence the richness of the sensory dimension, which may ultimately contribute to the enhancement of the user’s sense of autonomy (56). In addition, interactivity may also increase older adults’ sense of autonomy and competence. For example, the voice assistant (VA) technology in some age-appropriate smart wearables such as smartwatches is physically invisible, it does not force the user to be physically present in a particular location, and it uses natural language to provide interaction, which makes it easier for older adults to use (57). Meanwhile, social engagement of older people using social media provided by age-appropriate smart wearable devices is conducive to helping them have a good emotional experience and increase their sense of relatedness, which in turn enhances life satisfaction and improves older people’s health. For example, by using WeChat and other applications in smart watches to carry out online social activities, such as daily chats with friends and relatives, video calls and WeChat steps and friend circle likes, older persons are able to obtain a higher level of social support and social connections, thereby enhancing their sense of identity, belonging and relatedness (55). Therefore, based on the above, we propose the following hypotheses:

H2a: Interactivity of age-appropriate smart wearables is significantly positively correlated with older adults’ sense of autonomy.

H2b: Interactivity of age-appropriate smart wearables is significantly positively correlated with older adults’ sense of competence.

H2c: Interactivity of age-appropriate smart wearables is significantly positively correlated with older adults’ sense of relatedness.

Finally, regarding the directiveness of age-appropriate smart wearable devices, the most important thing is the directiveness of the user interface (58). In the past, due to the low digital literacy of older adults, they encountered various difficulties in using smart devices, coupled with the fact that they did not consider modern information technology to be essential in their daily lives, leading to a low acceptance and utilization rate among older adults. The reason for this problem is also inextricably linked to the directiveness design of the operating interface, and some irrational interface designs in the past have created a huge psychological burden for older adults (59). Nowadays, the optimized design of the interfaces of smart devices such as age-appropriate smart wearables has greatly increased the confidence of older people in using smart devices, such as the Tangible User Interface (TUI), which is a user interface that couples digital information with everyday physical objects and architectural surfaces, and which was initially designed to enhance the interaction between humans and digital information (60). This proprietary interface can help older adults better interact with the digital world through everyday physical objects by utilizing interface-guided design and other features to provide more intuitive data content and enhance their sense of autonomy and competence (60). Cho et al. evaluated the effect of Perceived User Interface Design (PUID) on users’ intention to continue using a self-paced e-learning tool, and found that PUID had a direct effect on perceived usefulness (61). This suggests that the guided design of the user interface of age-appropriate smart wearable devices enhances the user’s sense of autonomy and competence, and ultimately increases the satisfaction of older users (62). In addition, it has been shown that the simultaneous use of interfaces with obvious and easy-to-understand guide signs and text may help older adults to achieve effective interactions with minimal new learning or reliance on prior knowledge, contributing to a greater sense of competence, relatedness (63). Based on the above, the following three hypotheses of this paper are derived:

H3a: Directiveness of age-appropriate smart wearables is significantly positively correlated with older adults’ sense of autonomy.

H3b: Directiveness of age-appropriate smart wearables is significantly positively correlated with older adults’ sense of competence.

H3c: Directiveness of age-appropriate smart wearables is significantly positively correlated with older adults’ sense of relatedness.

### 3.2 The psychological needs and well-being of older adults

Ryan et al. state that every individual must satisfy three psychological needs of autonomy, competence, and relatedness if they are to protect their mental health and optimal human functioning (18). According to Baard et al. (64), people with higher satisfaction of these three needs tend to have positive attitudes towards target behaviors and are more likely to exhibit positive behaviors. At the same time, Baard et al. also noted that personal well-being also rises with their satisfaction with the three psychological needs.

Sense of autonomy, which encompasses the ability to take action and make self-choice, plays an important role in an individual’s perception of the state of the self at the physical, psychological, social, and spiritual levels, and is intrinsically linked to people’s sense of well-being in life. In short, the increased sense of autonomy brought about by age-appropriate smart wearables improves well-being (65). Additionally, age-appropriate smart wearables can help older adults enhance their sense of competence and positive self-supporting thoughts, thereby increasing their life well-being (66). It has been shown that older adults who do not interact with friends each month are twice as likely to develop depression. Older adults place a high value on emotional satisfaction and seek to minimize their emotional risks (67). Therefore, face-to-face online social interactions with family, friends, and other familiar people, facilitated by age-appropriate smart wearables, are especially necessary, and can provide a number of benefits to older adults, such as avoiding social isolation, providing emotional and social relatedness, sharing knowledge about health, offering social support, and buffering against physical and emotional stress, a sense of relatedness that is critical to alleviating depression in older adults. This sense of relatedness is essential for alleviating depression in older adults and even increasing well-being in old age (68). Based on the above arguments, the following hypotheses are proposed:

H4a: Sense of autonomy is significantly positively correlated with older adults’ well-being.

H4b: Sense of competence is significantly positively correlated with older adults’ well-being.

H4c: Sense of relatedness is significantly positively correlated with older adults’ well-being.

### 3.3 The mediating role of psychological needs

As mentioned earlier, the visibility of age-appropriate smart wearable device technological affordance can help enhance older people’s sense of autonomy, competence, and relatedness through data visualization and operation interface visualization (48, 52), and its interactivity can help enhance older people’s sense of autonomy, competence, and relatedness through human-computer interaction and human-to-human social interaction (55–57), and the directiveness of the technological affordance of age-appropriate smart wearable devices can also help to enhance older people’s sense of autonomy, competence, and relatedness through guided interface design (60–63). In addition, based on the above, we can also find that the psychological needs of older people for a sense of autonomy, competence, and relatedness can enhance the well-being of older people (64–68). In summary, the technological affordance of age-appropriate smart wearable devices can establish a close relationship with older adults’ well-being through its connection with their psychological needs for autonomy, competence, and relatedness. Therefore, we derive the following hypotheses:

H5a: Sense of autonomy mediates the relationship between technological affordance of age-appropriate smart wearables and well-being of older adults.

H5b: Sense of competence mediates the relationship between technological affordance of age-appropriate smart wearables and well-being of older adults.

H5c: Sense of relatedness mediates the relationship between technological affordance of age-appropriate smart wearables and well-being of older adults.

**Figure 1:**
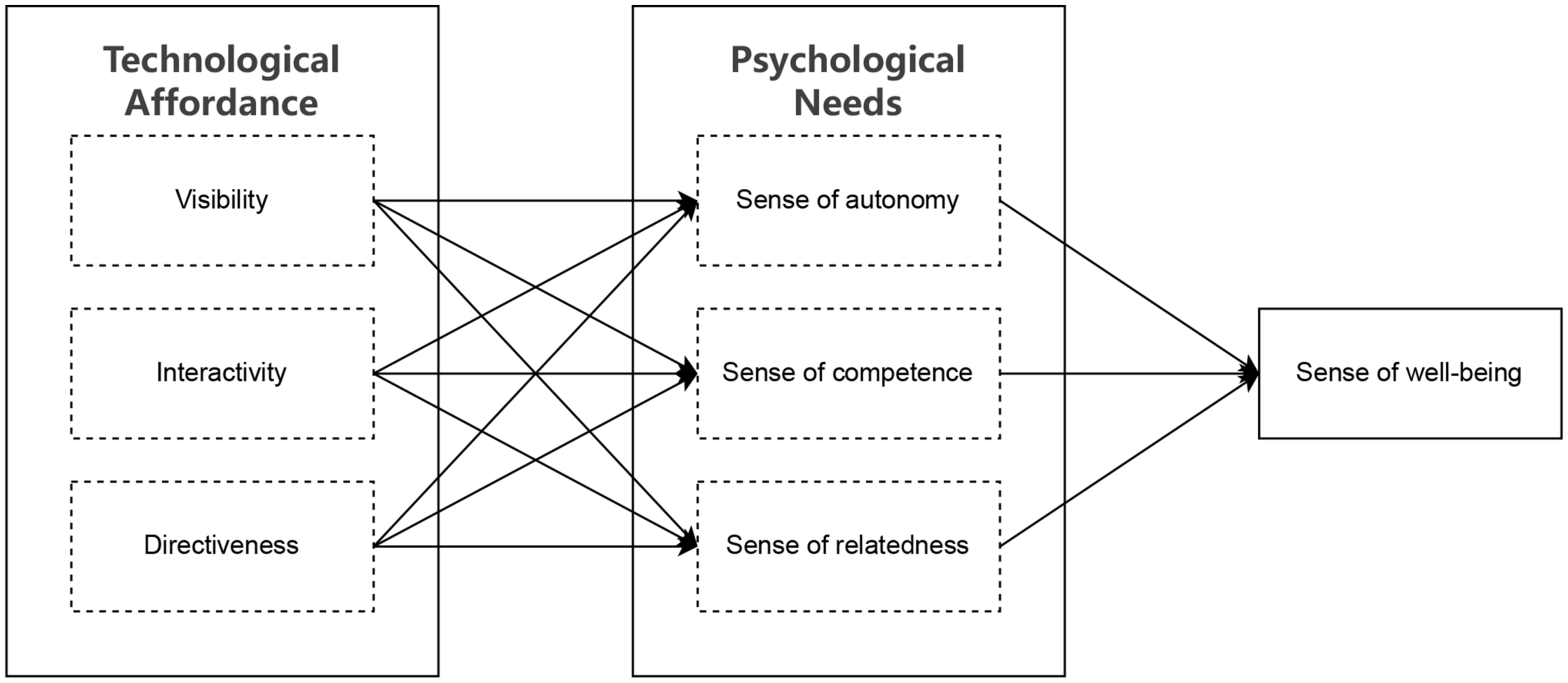
Research model.

Based on the above assumptions, we established the research model of this paper. The specific research model is shown in Figure 1.

## 4 Methods

### 4.1 Research design

This study used a quantitative research method to investigate the relationship between the technological affordance of age-appropriate smart wearable devices and the well-being of older adults. Data were collected through an online questionnaire using a 5-point Likert scale (1 = Strongly Disagree, 2 = Disagree, 3 = Fairly Agree, 4 = Agree, 5 = Strongly Agree) to measure relevant variables. The survey was conducted on February 22, 2025 through the online questionnaire platform “Wenjuanxing,” which is widely used in academic and market research in China for its reliability and ease of use. The questionnaire was divided into two main sections. The first part collects demographic information, including age, gender, education level, monthly income, and length of time using age-appropriate smart wearable devices. The second section focuses on measuring the study variables, including the visibility, interactivity, and directiveness of the device’s technological affordance, as well as the three psychological needs of older adults in the categories of autonomy, competence, and relatedness, and their overall sense of well-being. The specific measurement questions of the questionnaire are shown in Table 1, with a total of 18 questions.

**Table 1:** Latent variables and measurement items.

Latent variables	Measurement items
Visibility (78, 79)	Age-appropriate smart wearables allow me to visualize data about my blood sugar, blood pressure, heart rate, exercise steps, and more.
	Age-appropriate smart wearables allow me to clearly see what is on the screen.
	Age-appropriate smart wearables enable me to make video calls.
Interactivity (80, 81)	Voice assistants for age-appropriate smart wearables can answer the questions I ask.
	Age-appropriate smart wearables can explain what I am currently doing.
	User communities on age-appropriate smart wearables help me interact with family and friends.
Directiveness (82)	The age-appropriate smart wearable prompts me when I am not operating correctly.
	The navigation design of age-appropriate smart wearables guides me to the right actions.
	The age-appropriate smart wearable reminds me of measurement precautions when using the health monitoring feature.
SA (83)	The age-appropriate smart wearable gives me the freedom to use all of its convenient features.
	Age-appropriate smart wearables can help me with self-health management.
SC (84, 85)	Age-appropriate smart wearables have made me more capable of accomplishing things that were previously difficult.
	Age-appropriate smart wearables reduce my dependence on others.
SR (86)	Age-appropriate smart wearables can increase my connection with others through step comparisons and health challenge activities.
	Age-appropriate smart wearables reduce my loneliness.
SW (87)	Age-appropriate smart wearables can help me improve my health and reduce health risks.
	Age-appropriate smart wearable devices can enrich my spiritual and cultural life.
	Age-appropriate smart wearables can help me reduce medical and nursing costs.

SA, sense of autonomy; SC, sense of competence; SR, sense of relatedness; SW, sense of well-being.

### 4.2 Sample and data collection

The data for the study were collected through the online questionnaire platform “Wenjuanxing,” which is limited to a specific group of older people aged 60 and above who use age-appropriate smart wearable devices. At the beginning of the questionnaire, all respondents (older adults aged 60 and above) were provided with a concise explanation of “age-appropriate smart wearable devices” to ensure a unified understanding of this core concept. Such devices refer to intelligent electronic devices that can be worn on the body or integrated into clothing. They are specifically optimized to accommodate the physiological characteristics (e.g., declining vision), psychological needs (e.g., desire for autonomy), and usage habits of older adults. Typical examples include smart emergency call bracelets, smartwatches with fall detection and large-font health monitoring functions, and anti-wandering locators with voice prompts (9). A total of 247 questionnaires were collected. After data screening and eliminating incomplete or obviously careless responses, 233 valid questionnaires were ultimately obtained, yielding an effective recovery rate of 94.3%. The sample size of 233 in this study was determined based on two scientific considerations. First, it meets the commonly recommended criterion in structural equation modeling (SEM) research: specifically, there should be a minimum of 10–20 observations per indicator, and with 18 indicators in this study, the required sample size ranges from 180 to 360. Second, analysis via G*Power 3.1 verified that this sample size ensures sufficient statistical power (≥0.80) to detect medium-sized effects (*f*^2^ = 0.15) among the key variables.

### 4.3 Methods of data analysis

Data analysis was performed using SPSS and SmartPLS software. Descriptive statistical analysis was first performed to summarize the basic characteristics of the sample. Subsequently, the reliability and validity of the measurement instruments were assessed to ensure data quality. Finally, structural equation modeling (SEM) was used to test the research hypotheses. SEM is applicable to this study because it allows for the simultaneous testing of the relationships between multiple latent variables in order to gain a comprehensive understanding of the complex interactions between technological affordance, psychological needs, and well-being.

The analysis process began with the assessment of the measurement model, using a confirmatory factor analysis (CFA) to assess the reliability and validity of the constructs. Combined reliability (CR) and average variance extracted (AVE) were calculated to ensure internal consistency and convergent validity. Subsequently, structural models were tested to examine hypothesized relationships between variables. The overall fit of the model was assessed by a variety of metrics including chi-square (*χ*^2^), root mean square error of approximation (RMSEA), comparative fit index (CFI), and Tucker-Lewis index (TLI). Additionally, the mediating effects of sense of autonomy, sense of competence, and sense of relatedness were examined using the Bootstrap methodology in SmartPLS, with a setup of 5,000 replicated samples to ensure the robustness of the results. This comprehensive approach to data analysis ensured the reliability and validity of the findings and provided a solid foundation for the study’s conclusions.

## 5 Results

### 5.1 Sample characteristics

A total of 233 valid questionnaires were collected in this study, and the age distribution of the sample was even, with 30.9, 34.8, and 34.3% aged 60–69, 70–79, and 80 and above, respectively, and a slightly higher proportion of females (55.4%). Education level is mainly middle school (48.9%). Monthly incomes were concentrated in RMB (Renminbi) 3,000–5,000 (48.1%) and RMB 5,000–8,000 (24.5%). Regarding the duration of device use, participants used the device for 1–3 months (38.6%) and 3–6 months (26.2%) in a larger proportion. Specific demographic information is shown in Table 2.

**Table 2:** Descriptive statistics of the sample.

Basic characteristic	Classifications	Frequency	Percentage
Age	60–69 years old	72	30.9%
	70–79 years old	81	34.8%
	80 years and above	80	34.3%
Gender	Male	104	44.6%
	Female	129	55.4%
Level of education	Primary and below	75	32.2%
	Middle school	114	48.9%
	High school/vocational school	17	7.3%
	Three-year college	20	8.6%
	Undergraduate and above	7	3.0%
Monthly income range	Less than 3,000 yuan	25	10.7%
	3,000–5,000 yuan	112	48.1%
	5,000–8,000 yuan	57	24.5%
	More than 8,000 yuan	39	16.7%
Hours of use of age-appropriate smart wearables	Less than 1 month	55	23.6%
	1–3 months	90	38.6%
	3–6 months	61	26.2%
	More than 6 months	27	11.6%

### 5.2 Measurement model

This study used a confirmatory factor analysis (CFA) to assess the validity of the measurement model. Table 3 demonstrates the Composite Reliability (CR) and Average Variance Extracted (AVE) values for each latent variable. The CR values for all latent variables are greater than 0.7, indicating good internal consistency of the model (69). In addition, all latent variables have AVE values greater than 0.5, indicating that each latent variable can effectively explain the variance of its corresponding measurement question item (70). Table 4 demonstrates the correlation matrix between the latent variables. Bold on the diagonal is the square root of the AVE, which was used to assess discriminant validity (70). The results showed that the square root of AVE for all latent variables was greater than their correlation coefficients with other latent variables, indicating good discriminant validity for each latent variable.

**Table 3:** Results of the analysis of validating factors in the survey.

Latent variables	Measurement items	CR	AVE
Visibility	Age-appropriate smart wearables allow me to visualize data about my blood sugar, blood pressure, heart rate, exercise steps, and more.	0.782	0.545
	Age-appropriate smart wearables allow me to clearly see what is on the screen.		
	Age-appropriate smart wearables enable me to make video calls.		
Interactivity	Voice assistants for age-appropriate smart wearables can answer the questions I ask.	0.777	0.538
	Age-appropriate smart wearables can explain what I am currently doing.		
	User communities on age-appropriate smart wearables help me interact with family and friends.		
Directiveness	The age-appropriate smart wearable prompts me when I am not operating correctly.	0.792	0.560
	The navigation design of age-appropriate smart wearables guides me to the right actions.		
	The age-appropriate smart wearable reminds me of measurement precautions when using the health monitoring feature.		
SA	The age-appropriate smart wearable gives me the freedom to use all of its convenient features.	0.772	0.629
	Age-appropriate smart wearables can help me with self-health management.		
SC	Age-appropriate smart wearables have made me more capable of accomplishing things that were previously difficult.	0.819	0.694
	Age-appropriate smart wearables reduce my dependence on others.		
SR	Age-appropriate smart wearables can increase my connection with others through step comparisons and health challenge activities.	0.806	0.676
	Age-appropriate smart wearables reduce my loneliness.		
SW	Age-appropriate smart wearables can help me improve my health and reduce health risks.	0.788	0.554
	Age-appropriate smart wearable devices can enrich my spiritual and cultural life.		
	Age-appropriate smart wearables can help me reduce medical and nursing costs.		

CR, composite reliability; AVE, average variance extracted; SA, sense of autonomy; SC, sense of competence; SR, sense of relatedness; SW, sense of well-being.

**Table 4:** Latent variable correlations.

Latent variables	Mean	SD	Visibility	Interactivity	Directiveness	SA	SC-1	SC-2	SW
Visibility	3.558	0.890	**0.738**						
Interactivity	3.491	0.913	0.595	**0.733**					
Directiveness	3.489	0.939	0.600	0.595	**0.748**				
SA	3.481	0.995	0.534	0.581	0.582	**0.793**			
SC	3.534	1.076	0.572	0.530	0.629	0.568	**0.833**		
SR	3.479	1.010	0.510	0.575	0.571	0.499	0.487	**0.822**	
SW	3.542	0.923	0.625	0.553	0.599	0.499	0.575	0.495	**0.744**

The square root of AVE is shown in bold on the diagonal; SD, standard deviation; SA, sense of autonomy; SC, sense of competence; SR, sense of relatedness; SW, sense of well-being.

### 5.3 Structural model

**Figure 2:**
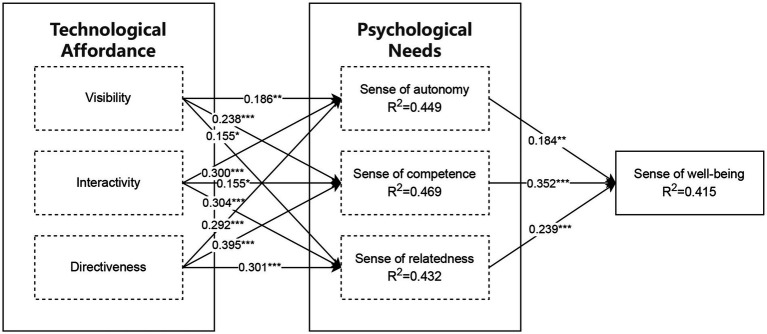
Results of the structural model. ***, **, and * indicate *p* < 0.001, *p* < 0.01, *p* < 0.05, respectively.

In this study, the theoretical model was tested using structural equation modeling (SEM) (see Figure 2 for the results of the structural modeling) to validate the path relationships between the latent variables. The results of the path analysis (e.g., Table 5) revealed that visibility were significantly and positively correlated with sense of autonomy (*β* = 0.186, *t* = 2.676, *p* < 0.01), sense of competence (*β* = 0.238, *t* = 3.490, *p* < 0.001), and sense of relatedness (*β* = 0.155, *t* = 2.053, *p* < 0.05), which supported Hypotheses H1a, H1b, and H1c; and interactivity were significantly and positively correlated with sense of autonomy (*β* = 0.300, *t* = 4.629, *p* < 0.001), competence (*β* = 0.155, *t* = 2.449, *p* < 0.05), and relatedness (*β* = 0.304, *t* = 4.517, *p* < 0.001) in support of hypotheses H2a, H2b, and H2c; and directiveness were significantly and positively correlated with sense of autonomy (*β* = 0.292, *t* = 4.759, *p* < 0.001), sense of competence (*β* = 0.395, *t* = 6.550, *p* < 0.001), and sense of relatedness (*β* = 0.301, *t* = 4.281, *p* < 0.001), supporting hypotheses H3a, H3b, and H3c. In addition, sense of autonomy (*β* = 0.184, *t* = 2.712, *p* < 0.01), sense of competence (*β* = 0.352, *t* = 5.884, *p* < 0.001) and sense of relatedness (*β* = 0.239, *t* = 3.996, *p* < 0.001) all were significantly and positively correlated with well-being, supporting hypotheses H4a, H4b, and H4c. The overall fit of the model was good, with good results in terms of chi-square (*χ*^2^ = 345.67, df = 120, *p* < 0.001), RMSEA (0.048), CFI (0.96) and TLI (0.95) were all at the desired level. The above findings provide empirical support for the theoretical model and lay an important foundation for subsequent studies.

**Table 5:** Summary of results from path analysis.

Hypotheses	Path relations	Coef	*t*-value	*p*-value	Conclusion
H1a	Visibility → SA	0.186	2.676	*p* < 0.01	Supported
H1b	Visibility → SC	0.238	3.490	*p* < 0.001	Supported
H1c	Visibility → SR	0.155	2.053	*p* < 0.05	Supported
H2a	Interactivity → SA	0.300	4.629	*p* < 0.001	Supported
H2b	Interactivity → SC	0.155	2.449	*p* < 0.05	Supported
H2c	Interactivity → SR	0.304	4.517	*p* < 0.001	Supported
H3a	Directiveness → SA	0.292	4.759	*p* < 0.001	Supported
H3b	Directiveness → SC	0.395	6.550	*p* < 0.001	Supported
H3c	Directiveness → SR	0.301	4.281	*p* < 0.001	Supported
H4a	SA → SW	0.184	2.712	*p* < 0.01	Supported
H4b	SC → SW	0.352	5.884	*p* < 0.001	Supported
H4c	SR → SW	0.239	3.996	*p* < 0.001	Supported

Coef, coefficient; SA, sense of autonomy; SC, sense of competence; SR, sense of relatedness; SW, sense of well-being.

### 5.4 Mediation effect test

For the mediating effect component, we tested the mediating roles of the sense of autonomy, competence, and relatedness between visibility, interactivity, and directiveness and well-being using the Bootstrap method in SmartPLS (5,000 repetitions of sampling), and the specific data are shown in Table 6. The results indicated that the sense of autonomy, competence, and relatedness played significant mediating roles in all three pathways. Specifically, visibility was significantly and positively correlated with well-being through the sense of autonomy [indirect effect coefficient = 0.034, 95% CI = (0.004, 0.080)], competence [indirect effect coefficient = 0.084, 95% CI = (0.030, 0.145)], and relatedness [indirect effect coefficient = 0.037, 95% CI = (0.001, 0.085)]; interactivity was significantly and positively correlated with well-being through the sense of autonomy [indirect effect coefficient = 0.055, 95% CI = (0.013, 0.111)], competence [indirect effect coefficient = 0.055, 95% CI = (0.013, 0.107)], and relatedness [indirect effect coefficient = 0.073, 95% CI = (0.030, 0.129)]; and directiveness was significantly and positively correlated with well-being through the sense of autonomy [indirect effect coefficient = 0.054, 95% CI = (0.013, 0.100)], competence [indirect effect coefficient = 0.139, 95% CI = (0.080, 0.203)], and relatedness [indirect effect coefficient = 0.072, 95% CI = (0.028, 0.127)]. The confidence intervals for all indirect effects did not contain 0, indicating a significant mediating effect (*p* < 0.05), supporting hypotheses H5a, H5b, and H5c. In addition, the direct relationships of visibility (*β* = 0.348, *p* < 0.001), interactivity (*β* = 0.179, *p* < 0.01), and directiveness (*β* = 0.285, *p* < 0.001) with well-being were also significant.

**Table 6:** Mediation effect test.

Path relations	Total effect coef	Direct effect coef	Total indirect effect		Indirect effect coef	95%CI
Visibility → SW	0.503***	0.348***	0.155***	Visibility → SA → SW	0.034	0.004 ~ 0.080
				Visibility → SC → SW	0.084	0.030 ~ 0.145
				Visibility → SR → SW	0.037	0.001 ~ 0.085
Interactivity → SW	0.361**	0.179**	0.182***	Interactivity → SA → SW	0.055	0.013 ~ 0.111
				Interactivity → SC → SW	0.055	0.013 ~ 0.107
				Interactivity → SR → SW	0.073	0.030 ~ 0.129
Directiveness → SW	0.550***	0.285***	0.265***	Directiveness → SA → SW	0.054	0.013 ~ 0.100
				Directiveness → SC → SW	0.139	0.080 ~ 0.203
				Directiveness → SR → SW	0.072	0.028 ~ 0.127

***, **, and * indicate *p* < 0.001, *p* < 0.01, *p* < 0.05, respectively; SA, sense of autonomy; SC, sense of competence; SR, sense of relatedness; SW, sense of well-being.

## 6 Discussion

The findings suggest that the visibility of age-appropriate smart wearable devices shows significant positive correlations with older adults’ psychological needs for autonomy, competence, and relatedness, and that the interactivity and directiveness of the devices are also significantly positively correlated with these psychological needs. This finding is consistent with previous research on the effects of digital technology on the quality of life of older adults, which also found that the intuitive and easy-to-use nature of technological devices can significantly enhance older adults’ sense of autonomy and social relatedness (71). In addition, we have found that these psychological needs are significantly positively correlated with the overall well-being of older adults. Past research has also pointed out that autonomy significantly enhances well-being by increasing older people’s control over their lives and their decision-making abilities, competence promotes well-being by increasing older people’s confidence in their abilities and their sense of accomplishment, and relatedness further enhances well-being by improving older people’s interactions with family, friends, and society (72). The study also validated the mediating roles of autonomy, competence, and relatedness between the technological affordance of age-appropriate smart wearable devices and the well-being of older adults. It was found that technological affordance is significantly positively correlated with older adults’ well-being through psychological needs.

Compared with previous studies, first, this paper not only expands the application of TAT in the older population (28), but also enriches the explanatory power of SDT, SOR theory, and SST in the field of smart aging. Second, this paper makes up for the neglect of mediating effects in existing studies (73), revealing the mediating role of psychological needs between technological affordance and well-being through empirical analysis, and providing a new theoretical perspective for understanding the connection between technology and older adults’ well-being. Finally, unlike previous studies that mainly focused on youth and general adult populations, this study focuses on the older population, while emphasizing the importance of considering the psychological needs of older adults in the design of age-adapted smart wearables, rather than just the functional needs (74).

Practically, this study offers critical insights for designing and developing age-appropriate smart wearable devices. First, the design of the device should focus on visibility and provide an intuitive data presentation and operation interface to enhance the sense of autonomy and competence of older adults. For example, existing research has demonstrated that age-appropriate smart wearable devices can effectively enhance physical activity levels among older adults by presenting health data digitally and graphically via visualization features, which helps them better understand their health status and facilitates self-monitoring. This approach has been shown to increase daily step counts (median: 1,312.23 steps) and moderate-to-vigorous physical activity duration (57.8 min per week) (75). Second, the interactive features of the devices should be further optimized to promote social interactions between older adults and others and enhance their sense of relatedness. For example, voice assistants and social apps in smartwatches can help older adults stay in touch with family and friends more easily (76). Similarly, empirical evidence demonstrates that interactive functionalities (e.g., social support systems and feedback mechanisms) represent a fundamental element of wearable device interventions, showing statistically significant improvements in both user participation rates and long-term adherence levels (75). Finally, the directiveness design of the device should be more humanized to help older people easily master the use of the device and lower the threshold of use. For example, through concise operation guides and real-time prompts, it can reduce the confusion and anxiety of older adults when using the device (77).

Although this study has achieved some valuable findings, there are still some limitations. First, it should be noted that the data were collected via an online survey platform (“Wenjuanxing”). This sampling approach may have resulted in a participant pool that was more familiar with digital technologies, potentially underrepresenting older adults with lower levels of digital literacy. Future research should therefore seek to validate these findings in community-based samples that include older adults with diverse digital literacy levels, particularly those who are less technologically adept, to enhance the generalizability of the results. Furthermore, the sample was mainly from China, and future research could be extended to older adults in other cultures to verify the generalizability of the findings. There may be differences in the acceptance of technology and usage habits of older adults in different cultures, and future cross-cultural studies could further explore the impact of these differences on the findings. Second, this study used cross-sectional data, and a longitudinal research design could be used in the future to further explore the long-term effects of technological affordance on the well-being of older adults. Longitudinal studies can help us better understand the causal relationship between technological affordance and well-being and whether this relationship changes over time. In addition, future research could further explore the role of other potential mediating variables in the relationship between technological affordance and well-being. For example, factors such as social support and technology anxiety may influence older adults’ experience and well-being with age-appropriate smart wearables. Future research could combine these variables to construct more complex theoretical models to more fully understand the relationship between technological affordance and older adults’ well-being.

## 7 Conclusion

This study empirically examines the relationship between technological affordance of age-appropriate smart wearable devices and the well-being of older adults, integrating insights from TAT, SDT, SOR theory, and SST. The findings demonstrate that visibility, interactivity, and directiveness are significantly positively correlated with older adults’ overall well-being through their positive associations with psychological needs—autonomy, competence, and relatedness. The mediating role of these psychological needs underscores the importance of designing wearable technologies that prioritize user-centered experiences to foster emotional and functional benefits for the aging population.

Theoretical contributions include extending the application of TAT to gerontechnology and bridging the gap between technical features and psychological well-being through SOR theory, SDT and SST. Practically, the study advocates for a “psychological needs-oriented” design framework, emphasizing intuitive interfaces, interactive functionalities, and guided usability to improve adoption and satisfaction among older users. Such innovations can transform smart wearables from mere functional tools into holistic well-being enhancers, aligning with global efforts toward healthy aging.

Despite its contributions, this study has limitations, including a geographically restricted sample and cross-sectional data. Future research should explore longitudinal and cross-cultural validations while incorporating additional mediators (e.g., social support) to refine the model. These advancements will further empower the development of inclusive, emotionally resonant technologies for aging populations worldwide.

## Data Availability

The original contributions presented in the study are included in the article/supplementary material, further inquiries can be directed to the corresponding author.
